# Transcriptomic Analysis of Differential Gene Expression in *Hevea brasiliensis* Under Short-Term Cold Stress

**DOI:** 10.3390/plants14182900

**Published:** 2025-09-18

**Authors:** Madushi Vishmitha Weeramange, Chenrui Gu, Shichao Xin, Xiaochuan Gu, Bin Yi, Tiandai Huang

**Affiliations:** 1 Rubber Research Institute, Chinese Academy of Tropical Agricultural Sciences, Key Laboratory of Biology and Genetic Resources of Rubber Tree, Ministry of Agriculture and Rural Affairs, Key Laboratory for Cultivation & Physiology of Tropical Crops of Hainan Province, State Key Laboratory Incubation Base for Cultivation & Physiology of Tropical Crops, Haikou Innovative Key Laboratory for Tropical Crop Seedlings, Haikou 571101, China; 2 National Key Laboratory of Tropical Crop Breeding, Sanya 572025, China; 3 College of Plant Science and Technology, Huazhong Agricultural University, Wuhan 430070, China

**Keywords:** *Hevea brasiliensis*, cold stress, transcriptome, gene expression

## Abstract

Cold stress limits the growth and productivity of *Hevea brasiliensis,* the primary source of natural rubber. This study investigated early transcriptomic responses in Reyan ‘7-33-97’ seedlings exposed to 4 °C, 10 °C, and 15 °C for 1, 2, and 4 h with room temperature (25 °C) as the control. RNA sequencing identified 9894 differentially expressed genes (DEGs), with the most significant transcriptional changes observed at 10 °C, indicating that genes to resist cold stress could not be mobilized at 4 °C, resulting in poor cold resistance of the rubber tree. KEGG enrichment analysis of DEGs between 10 °C (2 h) and 4 °C (2 h) revealed that genes involved in tryptophan metabolism (*HbKynL.x1*, *HbKynL*, *HbCLP1*, *HbCLP2*) and carbon metabolism (TCH4, XTH23), which contribute to cell wall modification, exhibited higher expression at 10 °C. Gene Ontology enrichment analysis highlighted significant involvement of “thylakoid,” “photosystems,” and “photosynthetic membrane,” alongside molecular functions such as “xyloglucan transferase activity” and “transcriptional regulator activity.” The interacting network of key pathways, including carbon metabolism (ko01200) and carbon fixation (ko00710) pathways, was sorted out, highlighting their integration with plant hormone signal transduction. Complex signaling networks, including MAPK, and kynurenine pathways coordinate the expression of cold-responsive genes and protective proteins, and it was confirmed and speculated that there is crosstalk response in cold defense mechanisms. Furthermore, 61 DEGs were associated with antioxidant processes, including major catalase and peroxidase enzymes. Our study shows that rubber trees physiological activities that respond to low-temperature signals cannot be carried out normally at 4 °C. The newly discovered metabolic pathway and the reason for abnormal cold signal transduction at low temperatures are the focus of future research on cold resistance.

## 1. Introduction

*Hevea brasiliensis* (Euphorbiaceae) is a tree native to the Amazon rainforest and is the primary source of natural rubber latex. Rubber cultivation is traditionally limited to tropical regions characterized by an average annual temperature of 28 °C ± 2 °C [1,2]. However, rising global demand for natural rubber has driven the expansion of rubber cultivation into non-traditional, cooler regions, exposing trees to sub-optimal temperatures [3]. The effects of low temperature can be categorized into chilling injury (0 °C–15 °C) and freezing injury (temperatures below 0 °C) [4,5]. These cold stresses may cause various detrimental symptoms in the plant body, including withering without leaf abscission, leaf drop, necrosis, interveinal chlorosis, bark discoloration, shoot dieback, and even death [5,6]. These results are from cell damage and retardation of metabolic, biological, and cellular functions, ultimately affecting the overall health and productivity of the trees [7,8].

Cold stress in *H. brasiliensis* activates specific signaling pathways and transcriptional programs that help the plant adapt and survive in harsh environments [9]. These responses include the detection of cold signals, their transduction into cellular responses, and the regulation of gene expression [10]. Low temperatures can reduce photosynthetic activity and carbon fixation and trigger the accumulation of protective metabolites such as soluble sugars and amino acids [11,12]. These metabolic adjustments are essential for maintaining cellular homeostasis and energy balance under stress. At the molecular level, cold stress activates complex signaling networks, including MAPK and ICE–CBF pathways, which coordinate the expression of cold-responsive genes and synthesis of protective proteins [13,14]. Phytohormones such as abscisic acid (ABA), indole-3-acetic acid (IAA), and ethylene play central roles in modulating cold stress responses in *Hevea*. ABA integrates multiple signaling pathways (ICE-CBF-COR, MAPK, and SnRK2) to enhance cold tolerance. While cold stress can initially trigger some ABA-independent responses, ABA amplifies and fine-tunes these pathways, ensuring robust plant adaptation to low temperatures [15,16]. Ethylene and IAA further regulate the stress adaptation by modulating gene expression and antioxidant defenses [7]. Cold-induced oxidative stress also leads to the differential expression of antioxidant genes, which help mitigate cellular damage by scavenging reactive oxygen species (ROS) [17]. Previous studies have identified 15 °C and 10 °C as critical thresholds for inducing physiological and molecular responses to cold in rubber trees, with short-term exposures (1–4 h) capturing the early dynamics of stress adaptation [6,18,19]. Despite these advances, the early transcriptional responses to chilling stress in the cold-sensitive Reyan ‘7-33-97’ clone remain poorly understood.

In this study, we used high-throughput RNA sequencing to profile gene expression changes in seedlings of the Reyan ‘7-33-97’ rubber clone subjected to various chilling temperatures and exposure durations. This rubber clone, extensively cultivated in China, is characterized by its high yield and resistance to cold and wind [20]. Our objectives were to characterize the short-term and low-temperature-dependent transcriptomic responses to cold stress, identify key biological processes and pathways involved in early cold adaptation, and discover candidate genes for improving cold tolerance in *Hevea brasiliensis.* The insights gained from this work will inform breeding strategies for enhancing cold resilience and support the sustainable expansion of rubber cultivation into cooler climates.

## 2. Results

### 2.1. RNA Sequencing Data Quality and Mapping

A total of 1,151,551,160 paired-end reads (150 bp) were generated from 30 samples, comprising biological triplicates subjected to cold stress treatments at 4 °C, 10 °C, and 15 °C, across three time points (1, 2, and 4 h) and RT (25 °C) using high-throughput RNA sequencing. Quality assessment revealed Q30 scores exceeding 96%, indicating high sequencing accuracy. Over 80% of the RNA-seq reads could be mapped uniquely to the reference genome, ensuring high-quality and reliable data for differential expression analysis. Transcript abundance was quantified using the FPKM method, enabling the identification of candidate genes involved in the cold stress response (Table S1).

### 2.2. Identification of Differentially Expressed Genes (DEGs)

**Figure 1:**
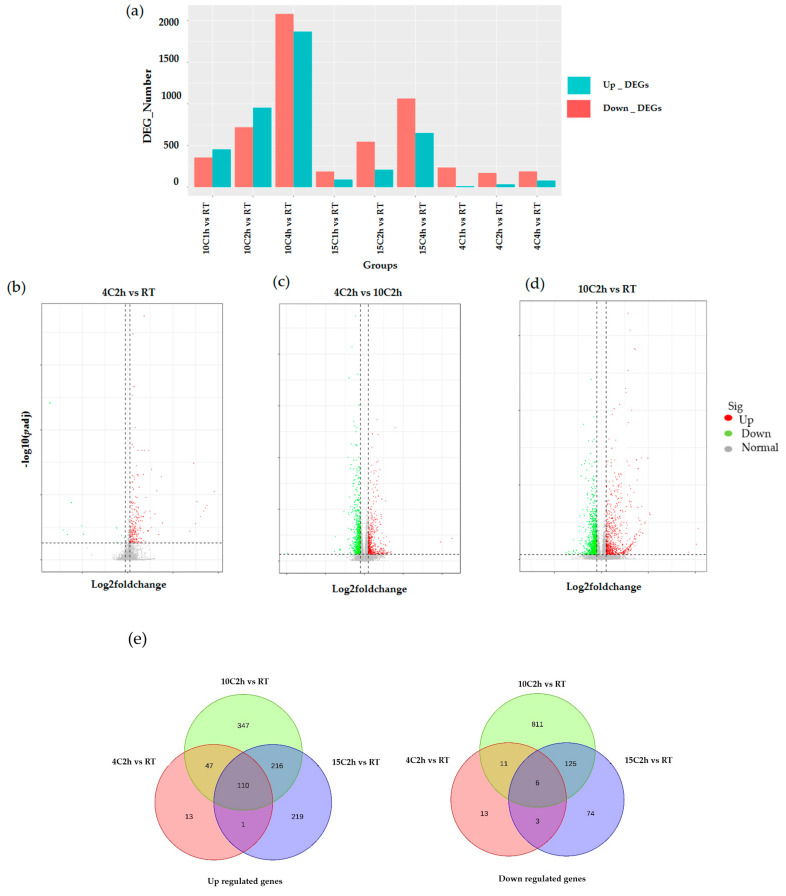
Differential expression gene analysis of various cold treatment conditions. (**a**) Bar plot of differentially expressed genes (DEGs) across different cold treatment conditions and sample collection in different time intervals. The x-axis represents the treatment groups, while the y-axis shows the number of DEGs. Red bars indicate the number of upregulated DEGs, and green bars indicate the number of downregulated DEGs. (**b**–**d**) Volcano plots showing log2 fold change vs. log10 *p*-value for three pairwise comparisons: 4 °C 2 h vs. RT, 4 °C 2 h vs. 10 °C 2 h, and 10 °C 2 h vs. RT. Each plot displays the distribution of genes based on their fold change in expression (x-axis) and statistical significance (y-axis). Green dots represent upregulated genes, red dots represent downregulated genes, and gray dots represent genes without significant changes. The dotted line indicates the threshold for statistical significance and fold change. (**e**) The left Venn diagram illustrates the upregulated genes at 4 h (4C2h), 10 h (10C2h), and 15 h (15C2h) of cold treatment. The right Venn diagram shows the downregulated genes at the same time points. Numbers indicate genes unique to each time point and those commonly regulated across different cold stress durations.

Differential expression analysis identified 9894 DEGs across all cold stress treatments compared to the control. The sample correlation was plotted in Figure S1. The number of DEGs varied by temperature and exposure time, with the highest number observed at 10 °C after 4 h (3948 DEGs; 2081 upregulated and 1867 downregulated), followed by 10 °C at 2 h (1673 DEGs; 720 up-regulated and 953 downregulated) (Figure 1a,c). In contrast, treatments at 4 °C resulted in fewer DEGs (Figure 1a,b), suggesting a delayed or attenuated transcriptional response at this lower temperature during short-term exposure. These results indicate that rubber seedlings mount a more rapid and sensitive transcriptional response to 10 °C than to 4 °C under short-term cold stress. To further investigate these differences, subsequent analyses focused on the 2 h exposure groups at 4 °C and 10 °C (1214 DEGs; 482 upregulated and 732 downregulated) (Figure 1d), through which we were able to identify an expanded set of genes associated with cold defense. Most of the genes that were upregulated at 4 °C are also included in the DEG set of 10C2h vs. RT and 15C2h vs. RT (47, 110, 1). The 10 °C treatment resulted in the highest number of upregulated genes (347) and downregulated genes (811), which are unique to the 10 °C treatment and not found in the gene lists obtained from the 4 °C or 15 °C treatments (Figure 1e, Table S6). Although the 15 °C treatment also produced a considerable number of differentially expressed genes, and there is a significant difference set compared to the 10 °C treatment, all the GO biological process terms enriched by these differential genes are entirely included in the enrichment results of the 10 °C treatment. Furthermore, biological processes such as ‘response to auxin’ or ‘response to hormone’ were not successfully enriched in the 15 °C treatment (Figure S2). This suggests that the 10 °C treatment can more intensively stimulate signaling pathways, thus evoking a more comprehensive cellular protective response. On the other hand, pathways associated with the maintenance of cellular metabolic homeostasis and mitigation of stress-induced damage, such as dioxygenase activity and ubiquitin-like protein transferase activity, were significantly enriched exclusively after 2 h of exposure to 4 °C (Table S7). The relatively lower enrichment observed following treatment at 10 °C suggests a less robust activation of protective gene networks compared to 4 °C. This pattern indicates that some injuries exist at 4 °C and might be more than 10 °C. Considering this, the data analysis strategy was decided to be changed. The 4 °C treatment was chosen as the control to exclude those genes responding to cell damage under the 10 °C treatment in order to retain the genes that actively protect cells, although room temperature (RT) served as the primary control for evaluating general cold stress responses. This approach enables clearer identification of gene expression patterns related to adaptive cold responses as opposed to those reflecting cellular damage at more extreme low temperatures.

### 2.3. Gene Ontology (GO) Enrichment

**Figure 2:**
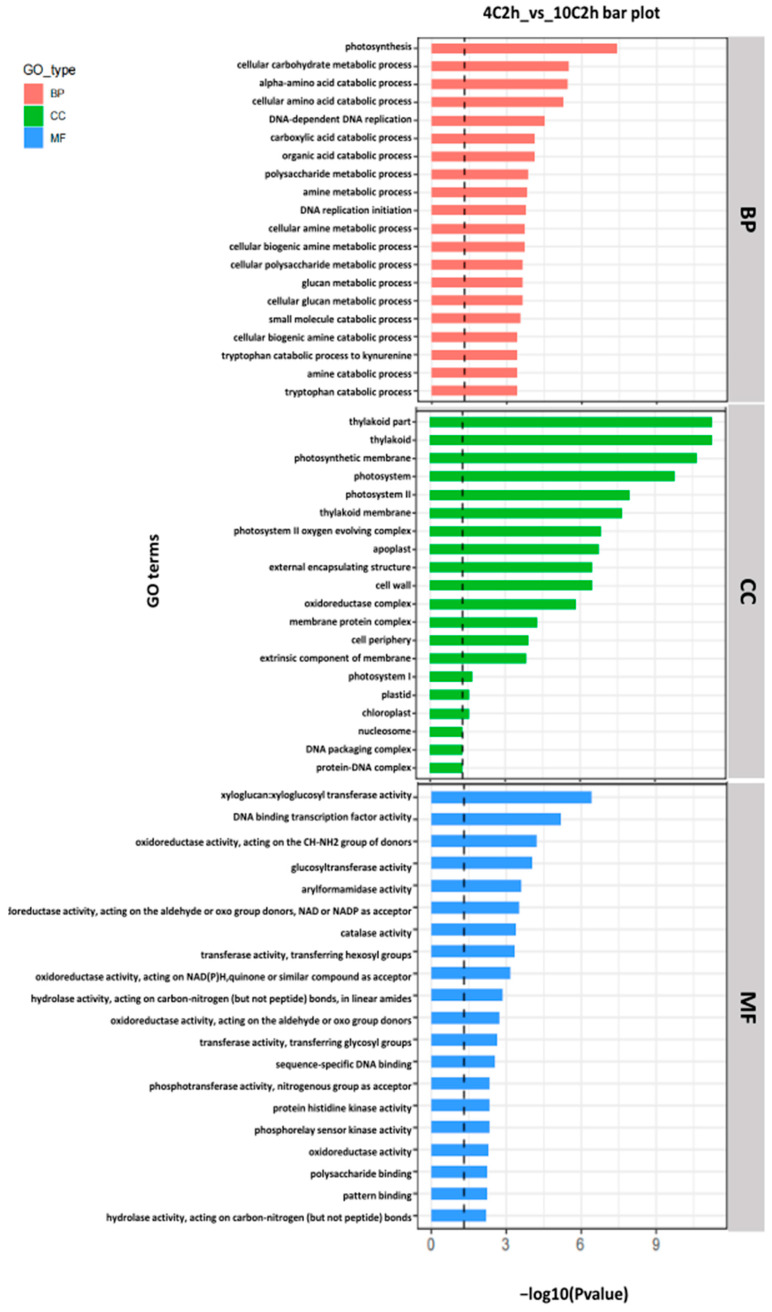
Gene Ontology (GO) enrichment analysis comparing 4 °C 2 h and 10 °C 2 h conditions. This bar plot illustrates the GO enrichment analysis results for three categories: Biological Process (BP), Cellular Component (CC), and Molecular Function (MF). The y-axis lists the GO terms with the name listed on their left side. The length of each bar corresponds to the −log10 (*p*-value).

A total of 1214 genes were annotated to explore the potential functions of differentially expressed genes. GO enrichment analysis comparing the 10 °C 2 h and 4 °C 2 h treatments revealed significant expression of terms related to photosynthesis, including “thylakoid,” “thylakoid part,” “photosystems”, “photosynthetic membrane”, and apoplast, cell wall, within the cellular component category. Biological process terms, such as carbohydrate metabolic process and oxidation-reduction process, were also enriched, reflecting metabolic adjustments under cold stress. Molecular function terms included xyloglucan transfer activity, DNA binding activity, and transcriptional regulator activity, indicating modulation of gene expression programs (Figure 2). These findings suggest that short-term (2 h) changes in biological processes, which are helpful for cold defense, are more pronounced in *Hevea* seedlings in response to 10 °C cold stress compared to 4 °C. Notably, pathways associated with the maintenance of cellular metabolic homeostasis and mitigation of stress-induced damage, such as dioxygenase activity and ubiquitin-like protein transferase activity, were significantly enriched exclusively after 2 h of exposure to 4 °C (Table S7). The relatively lower enrichment observed following treatment at 10 °C suggests a less robust activation of protective gene networks compared to 4 °C. This pattern indicates that although *Hevea* seedlings mobilize some protective responses at 4 °C, the associated GO terms also indicate cellular injury, which was greater than that observed at 10 °C.

### 2.4. KEGG Pathways and Enrichment Analysis

**Figure 3:**
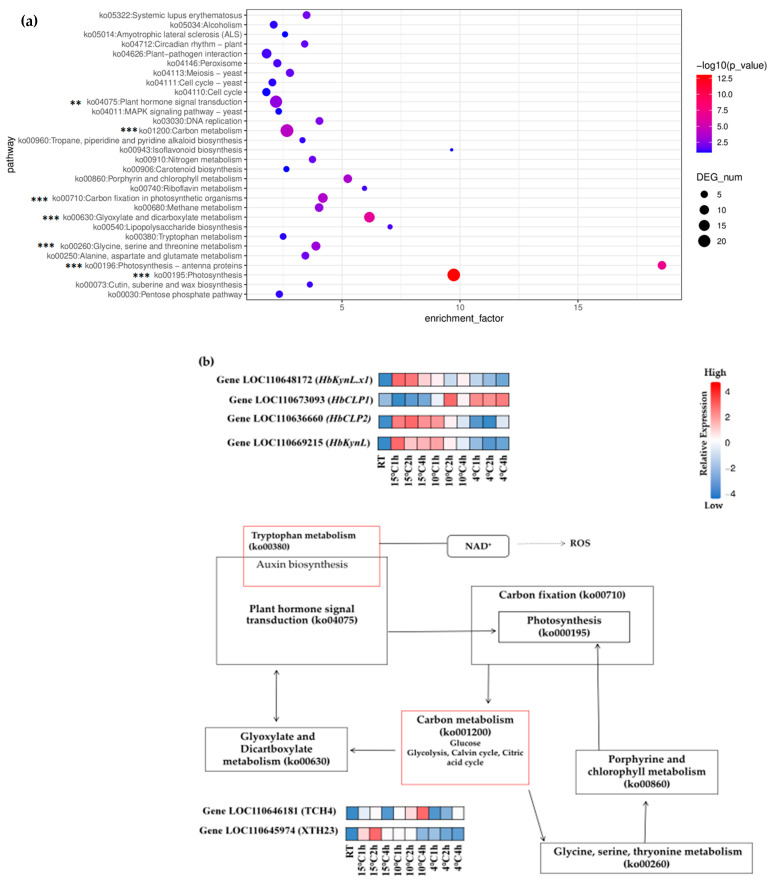

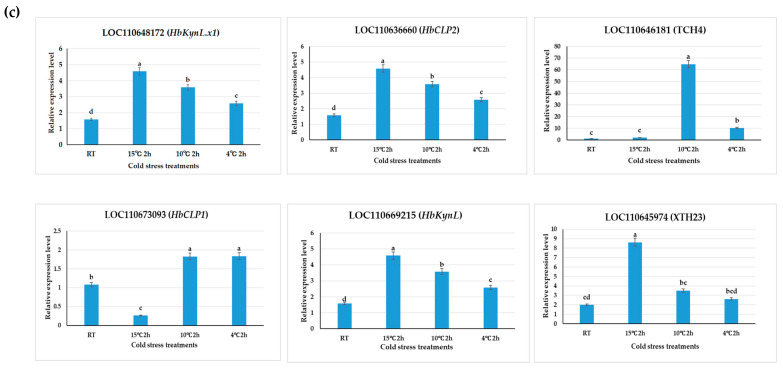
KEGG Enrichment Analysis of Differentially Expressed Genes (DEGs) in key pathways. (**a**) Enrichment bubble plot of DEGs. The x-axis of the bubble plot represents the enrichment factor. The y-axis represents pathways. The color represents the significance of enrichment −log10 (*p*-value) and asterisks (“**”, *p* < 0.05; “***”, *p* < 0.01) indicate significant differences as determined by the p-values from the KEGG enrichment analysis for pathways. (**b**) Interaction network of significantly enriched pathways (*p* < 0.01). The boxes represent KEGG pathways. ko00680 (methane metabolism) and ko03030 (DNA replication) without interaction with others were ignored here. Boxes with red frames were selected as key pathways that are essential to resist cold stress. Heatmaps at the top show the expression levels of DEGs involved in (tryptophan metabolism) ko00380; heatmaps at the bottom show the expression levels of DEGs involved in (carbon metabolism) ko001200. The abscissa of heatmaps represents treatments (RT, 15 °C 1 h, 15 °C 2 h, 15 °C 4 h, 10 °C 1 h, 10 °C 2 h, 10 °C 4 h, 4 °C 1 h, 4 °C 2 h, 4 °C 4 h). The arrows represent interactions between pathways, with the source pathway providing metabolites or signals to the target pathway. (**c**) Bar plots of relative expression levels for selected genes under cold stress treatments. The y-axis represents the relative expression levels, and the x-axis shows different cold stress treatments (RT, 15 °C 2 h, 10 °C 2 h, 4 °C 2 h). The error bars represent the standard deviation of replicates within the group. The significance of different cold stress treatments was further confirmed by Duncan’s test, which indicated simple letters on the error bars.

KEGG pathway analysis identified significant enrichment of pathways among all differentially expressed genes (DEGs) in the comparison groups (10 °C for 2 h vs. 4 °C for 2 h), including photosynthesis (ko000195), carbon metabolism (ko01200), carbon fixation (ko00710), and plant hormone signal transduction (ko04075) (Figure 3a). Among these, photosynthesis was a major pathway that was significantly enriched, demonstrating that low temperatures have a profound effect on it. Changes in the external environment, first detected by plants, trigger signals that lead to cold defense mechanisms. Under low-temperature stress, plant hormone signaling (ko04075) is significantly enriched and alters biological and mechanical processes. Notably, plant hormone signaling and MAPK signaling pathways were enriched, suggesting their involvement in cold defense responses and complex stress signaling crosstalk. This indicates that cold stress strongly affects primary metabolic and signaling processes, particularly those related to energy production and hormonal regulation. The heatmaps display the relative expression levels of selected candidate genes (LOC110648172 [*HbKynL.x1*], LOC110673093 [*HbCLP1*], LOC110636660 [*HbCLP2*], LOC110669215 [*HbKynL*], LOC110646181 [TCH4], and LOC110645974 [XTH23]) across different cold treatments and time points. These genes are mapped onto a pathway diagram that connects tryptophan metabolism, auxin biosynthesis, plant hormone signaling, photosynthesis, carbon metabolism, and glyoxylate/dicarboxylate metabolism. The diagram highlights the central role of these genes in mediating metabolic adjustments, hormone signaling, and stress responses during cold defense (Figure 3b). The bar graphs (Figure 3c) show qRT-PCR results validating the expression patterns of the same candidate genes under different cold stress treatments. The expression levels are consistent with the transcriptome data, confirming that genes involved in tryptophan metabolism (ko00380) (*HbKynL.x1*, *HbKynL*), chlorophyll metabolism (*HbCLP1*, *HbCLP2*), and cell wall modification (TCH4, XTH23) are differentially regulated in response to cold.

### 2.5. Regulation of Carbon Metabolic Pathways Under Cold Stress in Hevea brasiliensis

Carbon metabolism is intricately linked to cold tolerance in plants. KEGG analysis also revealed that carbon metabolism (ko01200) is regulated by 23 DEGs and the modulation of 11 DEGs in carbon fixation (ko00710) or amino acid metabolism (ko0830). Even though the *p*-value of these pathways has not reached a statistically significant level, these DEGs should not be ignored due to their intricate linkage to cold tolerance in plants that may favorably contribute to the cold tolerance of rubber trees [21] (Table S2).

### 2.6. DEGs Involved in Phytohormone Signaling Transduction Under Cold Stress in Hevea brasiliensis

**Figure 4:**
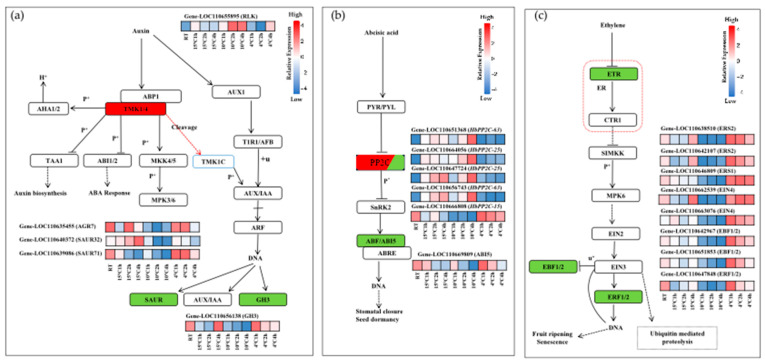
Key plant hormone signaling pathways and their regulation by DEGs. The arrows represent positive effects on activation, extrusion, or binding, while the ‘T’-ended arrow represents negative effects. Dashed arrows represent entry into other biological processes. Squares with rounded corners represent protein or protein family. Red or green fill of squares represents up-regulation or down-regulation of the gene/genes that code the family of proteins. The heatmaps adjacent to each gene represent the relative expression levels of (**a**) the auxin signaling pathway. The red arrow represents release by cleaving. The square with the blue border represents cleaving product. An arrow with a line segment perpendicular to it represents multiple regulatory effects of different members in the AUX/IAA protein family. (**b**) Abscisic Acid (ABA) Signaling Pathway. (**c**) Ethylene Signaling Pathway. The red dashed square represents endoplasmic reticulum (ER).

Plants must efficiently perceive and transduce cold stress signals to adapt and survive under low temperature conditions. In *Hevea brasiliensis*, the perception and signal transduction of cold stress are mediated by a set of 20 key differentially expressed genes (DEGs) identified through analysis of the KEGG pathway map04075. Among these DEGs, 5 genes were significantly upregulated, whereas 15 genes were downregulated when comparing chilling treatments at 10 °C for 2 h versus 4 °C for 2 h. These expression changes highlight the complex regulatory mechanisms involved in cold stress responses in *Hevea brasiliensis* and suggest specific molecular components that may contribute to chilling tolerance. Auxin signaling is initiated by external signals that trigger receptor-like kinase (RLK) proteins. In this study, the transmembrane kinase (TMK1/2) or receptor-like kinase (LOC110655895) protein was significantly upregulated, while other SAUR family members, such as LOC110640372 (SAUR32), LOC110639086 (SAUR71), LOC110635455 (ARG7), and LOC110656138 (GH3), were downregulated with the treatment of 10 °C compared to 4 °C, indicating a suppression of auxin biosynthesis and signaling under low-temperature conditions (Figure 4a). The ABA signaling pathway (Figure 4b), with a focus on the PP2C and ABF/ABI5, was induced under cold stress. The results indicate that type 2 protein phosphatases (PP2C) isoforms, such as LOC110651368 (*HbPP2C-63*), LOC110656743 (*HbPP2C-63*), LOC110664056 (*HbPP2C-25*), and LOC110647724 (*HbPP2C-25*), were upregulated, while LOC110666808 (*HbPP2C-15*) was downregulated at 10 °C for 2 h. LOC110669809 (ABI5), a bZIP transcription factor, was downregulated at 10 °C for 2 h but upregulated again at 4 °C for 2 h. These results suggest that ABA signaling is dynamically regulated in response to cold. Most genes involved in ethylene signaling exhibited differential expression. In this study, ethylene receptor genes, including LOC110638510 (ERS2), LOC110642107 (ERS2), LOC110646809 (ERS1), and LOC110662539/LOC110663076 (EIN4), were downregulated at 10 °C for 2 h. Genes are involved in both ethylene signaling and the MAPK signaling pathway, suggesting their dual roles in modulating cold stress responses. The induction of EBF1/2 and ERF1/2 indicates activation of ethylene-mediated transcriptional responses, which are involved in senescence and stress adaptation in usual plants [22]. Ethylene perception begins at the endoplasmic reticulum (ER), denoted by the red dashed square with ethylene receptors (ETR) in Figure 4c. Within the brassinosteroid signaling pathway, genes such as LOC110646004 (XTH23), LOC110646175 (XTH23), and LOC110646181 (XTH23), which are involved in cell wall modification, are upregulated under cold stress. This study observed the upregulation of the Cyclin-D3 gene (LOC110645095), suggesting its potential involvement in modulating the cell cycle to help the plant cope with cold stress in Figure S3. When plants are subjected to abiotic stress, numerous families of transcription factors are known to play a vital role in signal transduction and transcriptional regulation. According to the transcriptome analysis data, the highest log_2_ (fold change value) (7.9) was observed in the upregulated gene (*DREB1A/HbCBF1*) LOC110641584 and the downregulated gene (ERF) LOC110647848, both of which belong to the AP2 domain. These genes encode ethylene response factors (ERFs), which are transcription factors expressed under cold stress. The gene LOC110650424 (*MYC2*), related to the jasmonic acid signaling pathway, belongs to the bHLH domain and is a transcription factor that responds to cold stress in rubber seedlings. The Zf-C2H2 domain gene (*ZAT10*) LOC110651654 is upregulated, while the F-box-like protein family gene (*EIN3*) LOC110651853 is downregulated, both functioning in transcriptional activity. (ABF/ABI5) LOC110669809 is a downregulated, cold-responsive bZIP transcription factor involved in the abscisic acid signaling pathway under chilling temperatures.

### 2.7. The Role of Different Protein Kinases in Cold Stress Tolerance in Hevea brasiliensis

Cold stress significantly impacts plant physiology, requiring complex molecular adaptations to maintain cellular homeostasis. This current study has identified 49 kinases in *Hevea brasiliensis* at chilling temperatures (10 °C for 2 h vs. 4 °C for 2 h) in Table S3. There were 13 Wall Associated protein Kinases (WAK), 22 Receptor Like Kinase proteins (RLK), 6 L-type lectin-domain-containing receptor kinases, 4 Probable inactive receptor kinases, 3 calcium-dependent protein kinases (CDPK), and a Mitogen-activated protein kinase kinase kinase (MAPKKK). All of these protein kinases are processed in protein phosphorylation, and some of the kinases, LOC110648172 (*HbKynL.X1*) and LOC110673093 (*HbCPL1*) are critical regulators of cold tolerance through their involvement in the kynurenine pathway, NAD^+^ biosynthesis, and reactive oxygen species (ROS) management (Figure 3b). This study provides evidence from molecular studies to elucidate their roles in cold signal transduction, metabolic reprogramming, and cold stress resilience.

### 2.8. Transcription Factors (TFs) Involved in the Response to Cold Stress in Hevea brasiliensis

Transcription factors are crucial in regulating plant mechanisms for cold stress. A total of 136 differentially expressed transcription factor genes were identified in this study under 4 °C for 2 h vs. 10 °C for 2 h. Cold stress treatments include high expression of transcription factors in the MYB (25), zf-RING 2 (19), AP2/ERF (16), WRKY (10), bHLH (10), zf-B box (6), GATA (5), F-box (5), zf-C_3_HC_4_ (5), GRAS (4), and bZIP (3) protein families in Figure S4. The expression of the WRKY gene family was considerably upregulated in all genes at 10 °C for both 2 and 4 h. Within the zf-RING 2 family, ten genes exhibited upregulation, whereas nine genes demonstrated downregulation. Nine genes in the MYB family were upregulated, whereas sixteen genes were downregulated. In the bHLH gene family, nine out of ten genes were downregulated, with the exception of one gene. Within the AP2/DREB protein family, the gene LOC110636142 was downregulated, whereas the other four genes from the same family exhibited upregulation. Among the sixteen genes of AP2/ERF, five were downregulated while eleven were upregulated (Table S4).

### 2.9. Differentially Expressed Transcription Factors That Regulate Antioxidants in the Cold Stress

Cold stress induces changes in the expression of differentially expressed genes (DEGs) involved in antioxidant mechanisms, which play crucial roles in cold tolerance. These antioxidant enzymes can limit the activation of ROS against plant cell injuries. Plants can eliminate free radicals by increasing antioxidant enzyme activity to improve the defense system, which terminally mitigates the effect of ROS on cells. We identified 61 DEGs involved in antioxidant mechanisms under cold stress, including major Catalase (CAT) and Peroxidase (POD) genes (Table S5). The expression of CAT genes (LOC110638723 [CATALASE isozyme 2 Like], LOC110660630 [CATALASE 2 Like]) was downregulated following cold stress. While the expression of three POD genes (LOC110652184 [PEROXIDASE 12 Like], LOC110652185 [PEROXIDASE 12 Like]) was also downregulated, and LOC110670691 (PEROXIDASE 31 Like) was upregulated after cold stress response in *Hevea brasiliensis*.

## 3. Discussion

Cold stress is a critical abiotic factor limiting plant growth, development, and productivity, as well as affecting geographical distribution [12]. Plants respond to cold exposure through complex regulatory networks involving the differential expression of numerous functional and regulatory genes [21]. However, as a tropical species, the rubber tree (*Hevea brasiliensis*) may display a distinct transcriptomic response to cold stress compared to different *Hevea brasiliensis* clones [23]. This complexity has hindered challenges in identifying specific functional genes related to cold tolerance in rubber. To elucidate the molecular mechanisms and discover candidate cold tolerance genes, we conducted RNA-seq-based transcriptomic profiling of *H. brasiliensis* under cold stress [24].

Our study revealed a dynamic transcriptomic response highly dependent on both temperature and duration of exposure. The highest number of differentially expressed genes (DEGs) was observed at 10 °C after 4 h, highlighting a rapid and sensitive genomic adjustment to moderate chilling (Figure 1a). Exposure to the more severe temperature of 4 °C for a short duration (1 h) resulted in a relatively low number of DEGs. One reasonable explanation is that short-term severe cold may not fully trigger defense and metabolic pathways, limiting observable transcriptomic changes. Additionally, 4 °C exposure might induce stress pathways that promote energy conservation or damage response rather than gene activation detectable within this time frame (0–4 h). This observation aligns with prior findings in other plants, where moderate chilling temperatures can act as priming signals, effectively activating cold-responsive pathways [25]. Our DEG identification employed stringent statistical criteria, including a false discovery rate (FDR) of less than 0.05 and a minimum fold change threshold of 2. These thresholds ensured robust detection of biologically meaningful expression changes. This supports the hypothesis that very low temperature exposure for a short duration may be insufficient for mobilizing protective mechanisms in rubber trees. Previous studies [20,23,26] have identified numerous transcription factors and regulatory genes differentially regulated under cold stress in *H. brasiliensis*, underscoring the complexity of the cold response network. Collectively, these findings highlight that both the timing and level of gene expression changes are crucial determinants of successful cold resilience in rubber trees.

While the study primarily focused on the transcriptional responses at 4 °C and 10 °C, the inclusion of 15 °C in the experimental design provides an opportunity to explore the threshold for cold stress activation in *Hevea brasiliensis*. Although not extensively discussed in the results, the data for 15 °C likely represent a milder stress condition, which may serve as a priming stimulus for cold adaptation. Previous studies in other tropical plants [27,28] suggest that temperatures around 15 °C can trigger early stress signaling without causing severe physiological damage, potentially activating preparatory mechanisms such as the accumulation of osmoprotectant or the upregulation of early response transcription factors like DREB1A/CBF1 [1,14]. In rubber trees, this moderate chilling temperature might induce partial mobilization of pathways such as photosynthesis adjustment or hormone signaling (Auxin, ABA, ethylene), as observed in the 10 °C treatment but to a lesser extent.

The absence of substantial DEGs at 15 °C in this study may suggest a subthreshold stress level, where metabolic and transcriptional changes are subtle yet biologically relevant for acclimatization. Functional enrichment analyses, including Gene Ontology (GO) and KEGG pathways, identified significant involvement of photosynthesis, carbon metabolism, and plant hormone signal transduction in the cold response. Components related to photosynthesis, such as thylakoid and photosynthetic membranes, and processes like carbohydrate metabolism and oxidation reduction reactions (Figure 2) were enriched. These findings highlight the vulnerability of the photosynthetic machinery to cold stress, consistent with previous studies showing low temperatures impair photosynthetic efficiency and carbon fixation, resulting in energy shortages plants must counteract [13,29]. The enriched DNA binding and transcription regulator activities emphasize the central role of the transcription factors that play a pivotal role in coordinating early cold stress responses, reflecting the complex crosstalk between metabolic and signaling pathways facilitating environmental adaptation. Interestingly, pathways like tryptophan metabolism, which converts tryptophan to kynurenine, are subsequently linked to NAD^+^ biosynthesis. In mitochondrial glycolysis, NAD^+^ plays a crucial role in the reactive oxygen species (ROS) production, also connecting metabolic shifts to hormone signaling, notably auxin biosynthesis. Carbon metabolism (ko001200) pathways contribute intermediates (glyoxylate and dicarboxylate metabolism, amino acid metabolism) sustaining photosynthesis and stress responses (Figure 3a,b). The more significant biological changes at 10 °C for 2 h compared to 4 °C suggest a temperature- and time-dependent threshold effect in rubber tree transcriptional regulation, adding nuance beyond earlier studies [20,30].

In Figure 3a, pathways are broadly relevant to plant biology and directly reflect metabolic and regulatory adjustments associated with the cold stress response in *Hevea brasiliensis*. However, notable exceptions include the yeast cell cycle, yeast meiosis, and yeast MAPK signaling pathways. These annotations commonly arise due to the presence of evolutionarily conserved protein domains shared between many eukaryotes, including plants and yeast. Homologous plant genes are sometimes annotated with yeast specific pathway labels during computational enrichment analyses, not because they execute yeast-specific functions, but rather because of shared protein motifs and domain architectures. Thus, the identification of these yeast-associated pathways in the rubber tree transcriptome primarily reflects underlying evolutionary conservation rather than actual functional participation in yeast-related processes within *Hevea brasiliensis*. We retain these yeast pathways in the results to suggest that the MAPK signaling pathway and the meiotic pathway are likely to play important roles in the rubber tree’s resistance to cold stress as well, but due to the unique nature of such genes in rubber trees, they cannot be significantly enriched within the plant category. Research on MAPK signaling-related proteins specific to tropical crops should be emphasized in subsequent cold resistance breeding efforts.

Plants used diverse mechanisms to mitigate cold stress, including cell wall remodeling that maintains structural integrity and mediates reactive oxygen species signaling [31,32,33]. Enzymes like xyloglucan endotransglucosylase/hydrolase (XET/XTH/TCH4) (k14504), involved in cell wall loosening and brassinosteroid biosynthesis (Figure S2), showed altered expression, suggesting their role in cell cycle and growth adjustment to cold [34,35]. Xyloglucan endotransglucosylase and their expansion contribute to cell wall loosening by disrupting hydrogen bonds between cellulose microfibrils and pectin [36]. Cold signal perception involves receptor-like kinases (RLKs), such as the upregulated leucine-rich repeat (LRR) protein CLAVATA1 (LOC110655895), which modulates kinase activity through phosphorylation [37]. Our data showed downregulation of auxin-responsive genes (GH3, SAUR families), indicating a complex auxin signaling modulation under cold. Auxin transport and signaling components, including AUX1, ABP1, TMK1/4, and downstream MAPK pathways, interact with abscisic acid (ABA) signaling, which is a known positive regulator of cold tolerance via the ICE–CBF–COR transcriptional cascade.

Contrary to expectations, several PP2C genes, negative ABA signaling regulators, were upregulated at 10 °C, suggesting that certain isoforms may have unique roles in rubber tree cold adaptation divergent from classical models. Generally, ABA-bound PYR/PYL receptors inhibit PP2s, releasing SnRK2s to activate ABFs and trigger ABA responses [38,39]. However, in this study it shows (Figure 4b) upregulation of PP2C genes LOC110656743, LOC110651368 (*HbPP2C-63*), LOC110664056, and LOC110647724 (*HbPP2C-25*) at 10 °C after 2 h, suggesting that certain PP2C isoforms may regulate cold adaptation independently, unlike usual plants. Ethylene signaling begins with the inhibition of its receptors and continues through a cascade involving CTR1, SIMKK, MPK6, EIN2, and EIN3, which regulate downstream transcription factors associated with senescence and fruit ripening. Ethylene acts as a negative regulator of cold tolerance [40], showing gene expression that varies with temperature. In this study, ethylene responsive genes such as ERS1, ERS2, EIN4, and EIN3-binding F-box protein were downregulated at 10 °C and upregulated at 4 °C (Figure 4c). This indicates that programmed cell senescence in *Hevea brasiliensis* is more likely to be triggered at lower temperatures. Additionally, ethylene negatively impacts cold tolerance through the MAPK pathway and the HbEIN3 transcription factor, highlighting its role in reducing cold stress resistance in this plant [41].

Transcription factors (TFs) play central roles by binding to particular cis-regulatory regions in target gene promoters in the molecular networks underlying cold stress responses. Our transcriptome analysis identified hundreds of differentially expressed transcription factors. These include WRKY TFs, which are significantly expressed below 10 °C and key regulators of plant stress responses. For example, *CsWRKY40* enhances cold tolerance in tea plants by regulating *CsFBXL13* expression [42]. Similarly, *BpWRKY* genes in *Bergenia purpurascens* [27] and *SMWRKY* genes in *Solanum melongena* [43] are differentially expressed under cold stress, positively influencing tolerance. *VaWRKY33*, upregulated by *VaERF092* in Amur grapes [44], and *AFWRKY33* in *Acer fabri* enhance cold tolerance by stabilizing membrane lipids by binding to the W-box element in the promoter of the lipid metabolism gene *GPAT6* [27]. *MYC*-type bHLH TFs directly induce CBF gene expression, enhancing cold tolerance [13,45]. The *DREB1A/CBF1* transcription factor (LOC110641584) exhibited the highest induction, binding *CRT/DRE* motifs to activate cold-responsive genes and improve survival, corroborating DREB1A’s known function in enhancing antioxidant enzymes and cold tolerance [46]. bZIP TFs such as *ABI5* also contribute to cold responses [47]. The identification of WRKY, AP2/DREB, and bHLH family transcription factors (TFs) (Figure S3) suggests conserved homology-based functions, but functional validation in rubber trees remains necessary to confirm specific roles in cold resilience.

Cold stress induces excessive accumulation of reactive oxygen species (ROS), causing oxidative damage [19], particularly in chloroplast components like thylakoids and photosynthetic membranes, leading to impaired photosynthesis [48]. Under normal conditions, ROS levels are tightly regulated; however, abiotic stress disrupts this balance, resulting in oxidative stress and cellular damage [49]. However, under abiotic stress, excess ROS can induce oxidative stress and cellular damage [50]. Genes involved in coping with the negative effects of cold stress led to the production of large amounts of ROS. Our transcriptomic data showed significant elevation of peroxidase (*POD*) and catalase (*CAT*) expression after cold stress. Peroxidase functions to clear ROS, with one *POD* gene upregulated and two downregulated, indicating a complex regulation to mitigate oxidative damage and enhance cold tolerance at 10 °C. Similarly, one *CAT* gene was upregulated while two were downregulated, suggesting modulation of H_2_O_2_ scavenging capacity to adapt to cold stress. *CAT* activity is associated with preventing oxidative damage by scavenging H_2_O_2_. Previous studies [51] demonstrated that *DREB1A* overexpression increases antioxidant enzyme activities, maintaining cell membrane stability under low-temperature stress.

Moreover, cold stress triggers metabolic shifts such as accumulation of soluble sugars and amino acid catabolism, which act as osmoprotectants and energy sources [52]. These changes stabilize cellular structures, maintain membrane integrity, and supply energy required during chilling conditions. The enrichment of genes involved in tryptophan catabolism and related pathways further supports metabolic reprogramming as a key component of cold defense response [28]. Soluble sugars reduce cell water potential, improving water retention and preventing protein coagulation caused by low temperatures. Sugars also provide carbon sources that induce other cold resistance-related physiological and biochemical processes, enhancing overall cold tolerance [53].

In summary, our integrated physiological and transcriptomic analyses provide comprehensive insights into the molecular and metabolic mechanisms underlying cold stress response in *Hevea brasiliensis*. The findings highlight the importance of temperature and exposure duration on gene expression dynamics, the critical roles of photosynthesis and hormone signaling pathways, cell wall remodeling, transcription factor regulation, ROS detoxification, and metabolic reprogramming in conferring cold tolerance. These results lay a foundation for future functional studies and genetic improvement of rubber trees for enhanced cold resilience.

## 4. Materials and Methods

### 4.1. Plant Material and Cold Treatment

Tissue culture test tube seedlings (Figure S5) of the rubber clone Reyan ‘7-33-97’ were grown under controlled conditions (cool white fluorescent light at 35 µmol m^−2^ s^−1^, a 6/8 h photoperiod and 65% relative humidity) and subjected to cold stress treatments in a climate-controlled growth chamber. Seedlings were exposed to chilling temperatures of 4 °C, 10 °C, and 15 °C, with room temperature as the control. Samples were collected at 1, 2 and 4 h after treatment initiation. Each treatment and time point included three biological replicates. Immediately after collection, leaf samples were frozen in liquid nitrogen and stored at −80 °C until RNA extraction.

### 4.2. RNA Extraction, Library Construction, and Sequencing 

Total RNA was extracted from frozen leaf tissues using the RNA Extraction Kit (Tiangen, Beijing, China). RNA quality and concentration were assessed using a NanoDrop 2000 spectrometer (Thermo Scientific, Waltham, MA, USA), with A260/A280 ratios between 1.8 and 2.1 considered acceptable. RNA integrity was confirmed by gel electrophoresis. RNA samples were sent to Wuhan Carbon Code Biotechnologies (Wuhan, China) for library construction and sequencing. Libraries were prepared following standard protocols and sequenced on the DNBSEQ-T7 platform, generating over 6 Gb of paired-end 150 bp reads per replicate. 

### 4.3. Sequence Data Processing and Differential Expression Analysis

Raw sequencing reads were quality filtered to remove adapters and low-quality bases. Clean reads were aligned to the *Hevea brasiliensis* reference genome [54] using Hisat2 (version 2.2.1) [55] with default parameters. Transcript assembly and quantification were performed using StringTie (version 2.2.3) [56]. Gene expression levels were normalized and expressed as fragments per kilobase of transcript per million mapped reads (FPKM). Differential gene expression analysis was conducted using the R package DESeq2 (https://bioconductor.org/packages/release/bioc/html/DESeq2.html), accessed on 19 October 2024 [57], which emphasizes a negative binomial generalized linear model to estimate significance based on replicate variance. Genes with expressions of *Padj* ≤ 0.01 and |log2FC| ≥ 1.0 were considered differentially expressed genes (DEGs). 

### 4.4. Quantitative Real-Time PCR (qRT-PCR) Validation

Total RNA was reverse transcribed into cDNA using the HiScript III All-in-one RT Super Mix Perfect or qPCR Kit (Vazyme, Nanjing Vazyme Biotech Co. Ltd., Nanjing, China). qRT-PCR was performed on an Applied Biosystems 7500 Fast Real-Time PCR System using ChamQ Universal SYBR qPCR Master Mix (Vazyme, Nanjing, China). Gene specific primers (Table 1) were designed based on RNA-seq data. Actin (ACT) was used as the internal reference gene, with its expression stability under cold stress confirmed in previous studies [58]. Each sample was performed in technical triplicates. Relative gene expression was calculated using the 2^−ΔΔCT^ method. Statistical significance was assessed by one-way ANOVA followed by Duncan’s multiple range test using SAS software (version 9.4) to evaluate the differences between group means, and bar plots were generated using SR plots to visually represent the data.

### 4.5. Gene Ontology (GO) Enrichment Analysis

GO enrichment analysis was performed using the Database for Annotation, Visualization, and Integrated Discovery (DAVID, Version 2023q4) [59] to elucidate the potential functions of differentially expressed genes (DEGs). Gene ontology (GO) analysis was conducted on DEGs in *Hevea brasiliensis* under various low temperature stress treatments. Among the total DEGs, the potential functions of the gene ontology distribution system for rubber genes were categorized into three main classes: biological process (BP), cellular component (CC), and molecular function (MF). Based on the significance of interactions across different cold stress treatments and time intervals, DEGs were screened by setting thresholds via classic Fisher and FDR (false discovery rate) values. In the comparison of the two groups, a classic Fisher value ≥ 0.01 and an FDR value ≥ 0.01 were considered as significant and differential expression.

### 4.6. Kyoto Encyclopedia of Genes and Genomes (KEGG) Pathway Analysis

KEGG pathway enrichment was conducted using DAVID to identify significantly affected metabolic and signaling pathways. DEGs were mapped to KEGG pathways, with significant enrichment defined by *p* < 0.05. An e-value cutoff of <1 × 10^−4^ was used for gene annotation.

## 5. Conclusions

This transcriptomic study delineates the temperature-dependent molecular mechanisms underpinning the early chilling stress response in *Hevea brasiliensis*. We demonstrate that a moderate chilling temperature of 10 °C elicits a more pronounced and coordinated transcriptional activation of defense pathways compared to a severe 4 °C treatment. The attenuated response at 4 °C, characterized by a significant reduction in differentially expressed genes (DEGs), suggests a critical failure in the mobilization of cold acclimation mechanisms, which fundamentally limits the cold resilience of rubber trees at critically low temperatures. Our integrated analysis reveals that the core early response involves the concerted regulation of photosynthesis (ko00195), carbon metabolism (ko01200), carbon fixation in photosynthetic organisms (ko00710), tryptophan metabolism (ko00380), and plant hormone signaling and transduction (ko04075), with key genes facilitating this metabolic adjustment (*HbKynL.x1*, *HbKynL*, *HbCLP1*, *HbCLP2*, *TCH4*, *XTH23*) being significantly upregulated at 10 °C. This response is orchestrated by a complex phytohormonal crosstalk, featuring dynamic regulation of ABA, auxin, and ethylene signaling pathways, which in turn modulate the expression of pivotal transcription factors such as *DREB1A/HbCBF1* and genes encoding antioxidant enzymes (CAT, POD) to maintain cellular homeostasis. Our findings posit that the incapacity to initiate integrated transcriptomic reprogramming at 4 °C is a principal determinant of cold sensitivity in *H. brasiliensis*. This study provides a comprehensive molecular framework and identifies a suite of candidate genes, offering valuable targets for future functional genomics research and breeding strategies aimed at enhancing cold tolerance.

## Figures and Tables

**Table 1:** Primer Sequences used for qRT-PCR validation.

Gene Name	Gene Id	Forward/Reverse Primer	Primer Sequence (5′ to 3′)
TCH4	LOC110646181	qLOC110646181-F	CGCATCCCATCTCCTCACTAC
		qLOC110646181-R	TGGCTGCCATGGAAGAGAAG
*DREB 1A/CBF1*	LOC110641584	qLOC110641584-F	GCGTCTAGTCATGAGGGCTG
		qLOC110641584-R	AATCCGAACCAAGACGAGAAT
*HbKynL*	LOC110669215	qLOC110669215-F	GGCTACTGATTGATGTGCCA
		qLOC110669215-R	TCTGAAATTTACCTGTCGGTG
*CLP1*	LOC110673093	qLOC110673093-F	CTTTCAGGACGCTGACAGAA
		qLOC110673093-R	TGGCACTTGCTATACTCTCAA
*CLP2*	LOC110636660	qLOC110636660-F	GATTCTTCCCTCTCCACAGC
		qLOC110636660-R	CGGTGATGGAATGTAGCAGA
*CLP2*	LOC110648172	qLOC110648172-F	CCTTTCTTGCCTCTCCACCAC
		qLOC110648172-R	CGGCGGACTGGGATTAGATT
XTH23	LOC110645974	qLOC110645974-F	CTTCCTCTGTTGGCTATTCAGC
		qLOC110645974-R	GGTTGTCACAGCTCTCAGGTATT

## Data Availability

All high-throughput sequencing data have been uploaded and published in the Bio-Project PRJCA042855 of China National Center for Bioinformatics (https://ngdc.cncb.ac.cn/gsa/browse/CRA027825, accessed on 14 July 2025).

## Supplementary Materials

The following supporting information can be downloaded at: https://www.mdpi.com/article/10.3390/plants14182900/s1, Table S1. High throughput Sequencing quality summary; Table S2. KEGG Enrichment Results (10C2h_vs_4C2h); Table S3. Protein kinases (10C2h_vs_4C2h); Table S4. Transcription factors (10C2h_vs_4C2h); Table S5. Antioxidant enzymes function in Hevea; Table S6. DEG treated samples vs. RT statistics.xlsx; Table S7. (DEG treated samples vs. RT) GO Enrichment.xlsx; Table S8. Heatmap of all DEGs with FPKM value.xlsx; Table S9. GO terms (10C2h_vs_4C2h).xlsx; Table S10. GO terms (15C2h_vs_4C2h).xlsx; Figure S1. Cold stress treatment samples correlation plot; Figure S2. Comparative Enrichment Analysis of Gene Ontology terms in Transcriptome under Cold stress treatments; Figure S3. Schematic diagram of Brassinosteroid signaling pathway. The brassinosteroid signaling pathway was mapped in Figure S3, highlighting key components such as BAK1, BRI1, BSK, BSU1, BIN2, and BZR1/2. Downstream, the pathway regulates genes associated with both cell division (CYCD3) and cell elongation (TCH4) by up regulated genes. Heatmaps next to each gene display their relative expression levels under different cold stress treatments; Figure S4. Heatmap of Transcription factors; Figure S5. Tissue cultured test tube seedlings of the rubber clone Reyan ‘7-33-97’. Pair-wise comparison of different cold treatments was represented through volcano plots in a presentation file.
