# Supplementary material for: Transcriptomic Analysis of Differential Gene Expression in Hevea brasiliensis Under Short-Term Cold Stress
Source: Plants (Basel). 2025 Sep 18;14(18):2900. doi: 10.3390/plants14182900 (PMC12473355; doi:10.3390/plants14182900)
Supplement: Supplementary file 1 [file plants-14-02900-s001.zip › Supplementary figures.pdf]

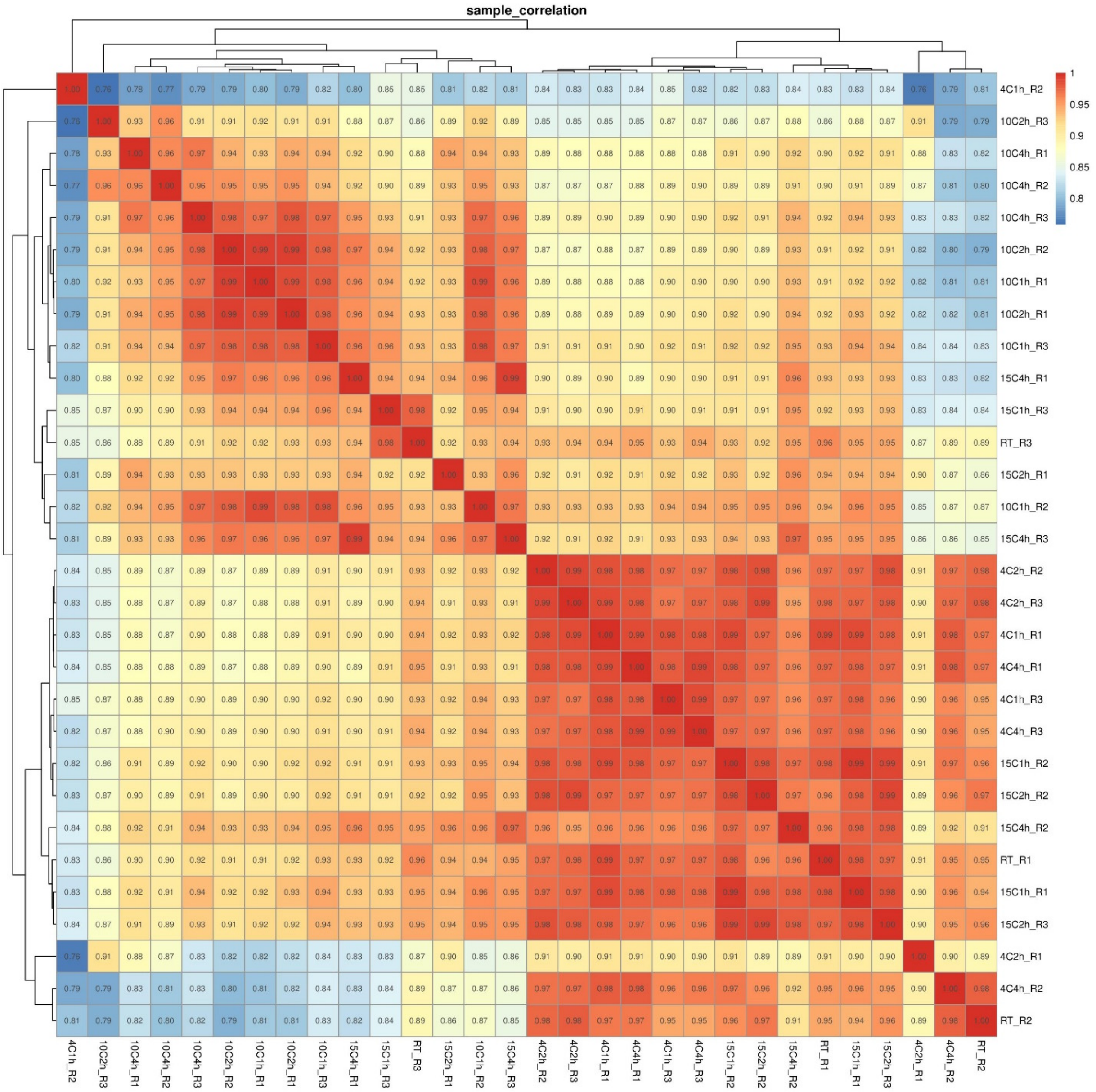

Figure S1. Cold stress treatment samples correlation plot.

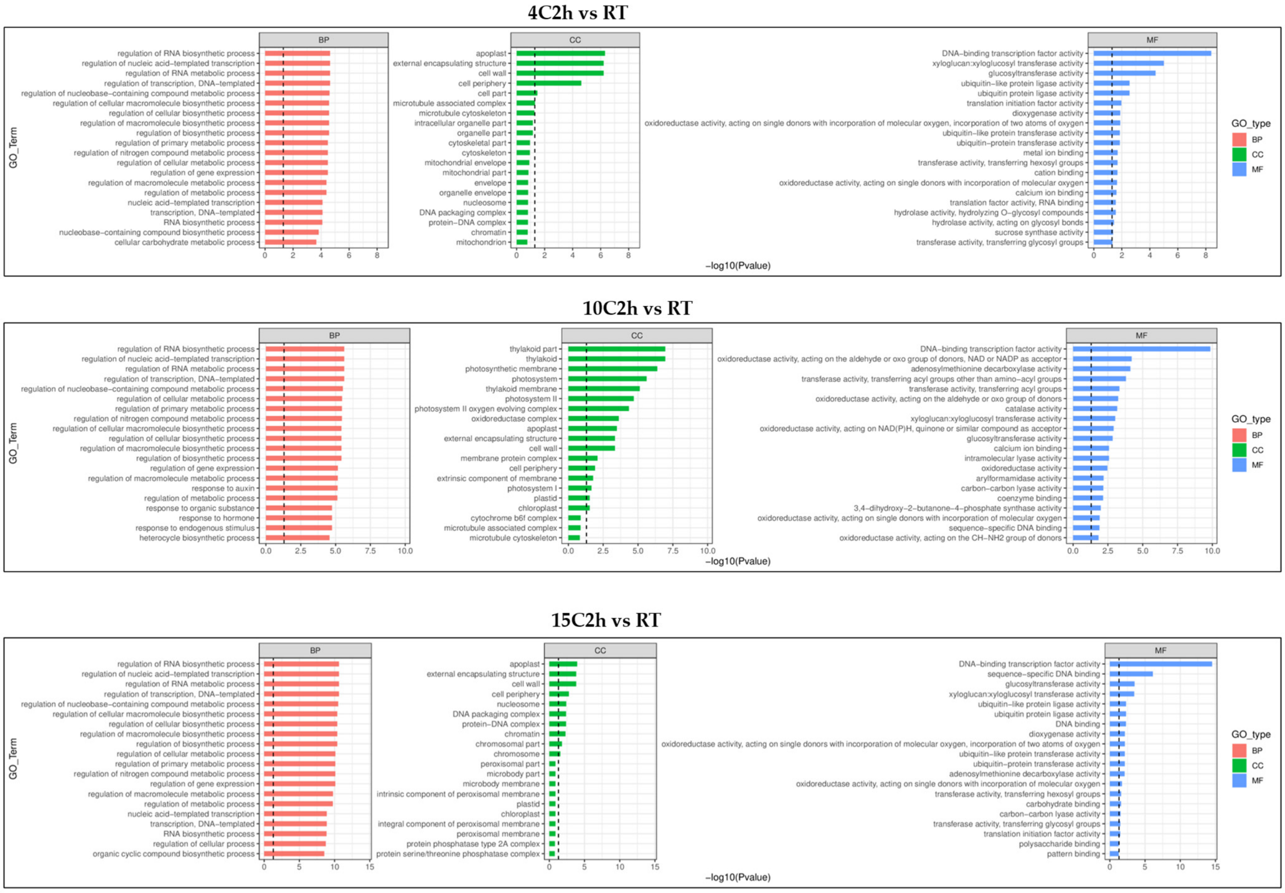

Figure S2. Comparative Enrichment Analysis of Gene Ontology terms in Transcriptome under Cold stress treatments.

Bar plots represent the top significantly enriched gene ontology (GO) categories across three pairwise comparisons of low temperature treatments versus room temperature controls (4 h vs. RT, 10 h vs. RT, and 15 h vs. RT). Enrichment is shown for Molecular Function (MF), Biological Process (BP), and Cellular Component (CC) categories with significance defined as $–\log_{10}$ *p*-value ≥ 2. The length of each bar corresponds to the magnitude of the –log10 p-value, indicating the strength of enrichment for each GO term.

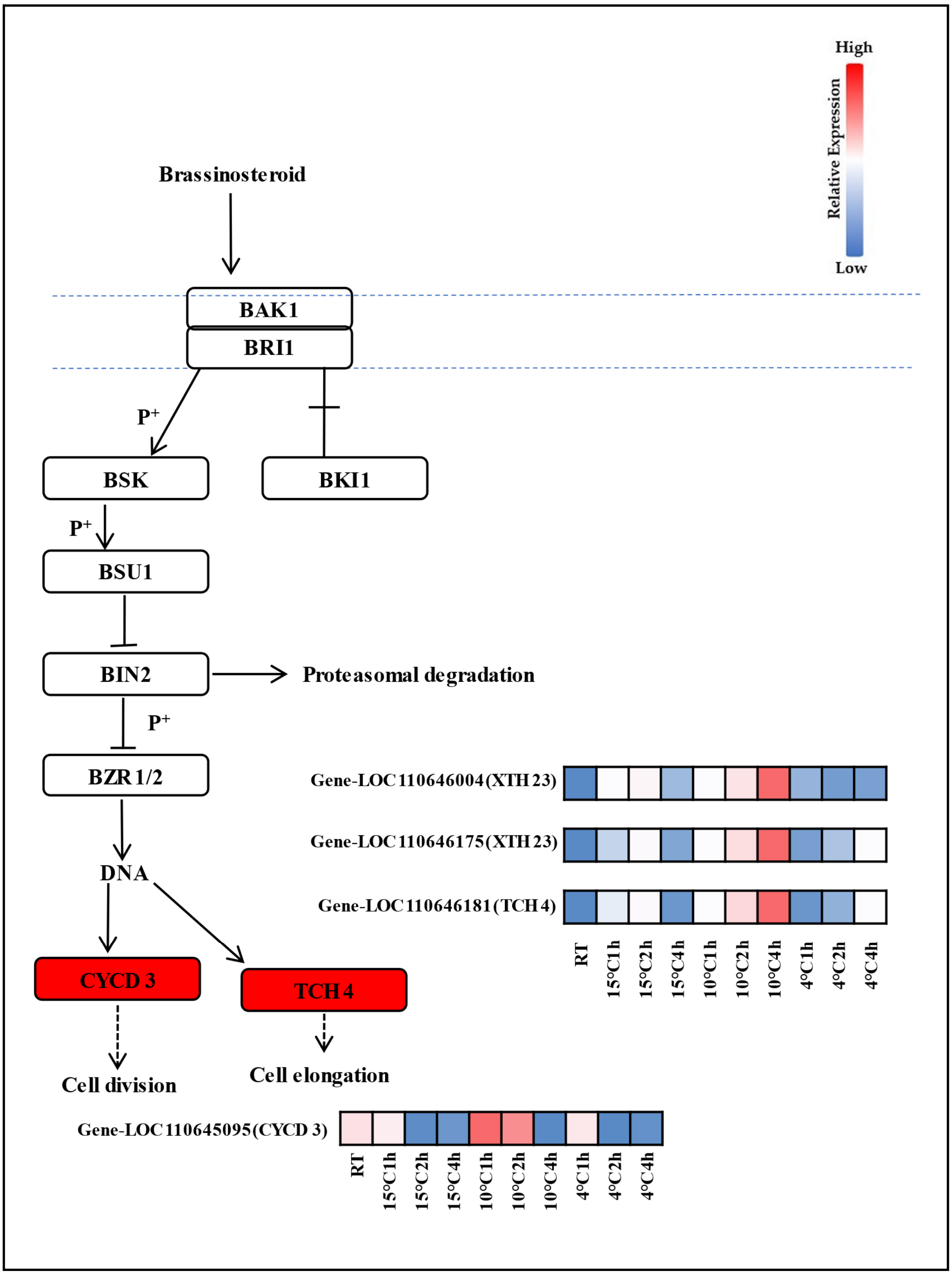

Figure S3. Schematic diagram of Brassinosteroid signaling pathway

The brassinosteroid signaling pathway was mapped in Figure S1, highlighting key components such as BAK1, BRI1, BSK, BSU1, BIN2, and BZR1/2. Downstream, the pathway regulates genes associated with both cell division (CYCD3) and cell elongation (TCH4) by up regulated genes. Heatmaps next to each gene display their relative expression levels under different cold stress treatments.

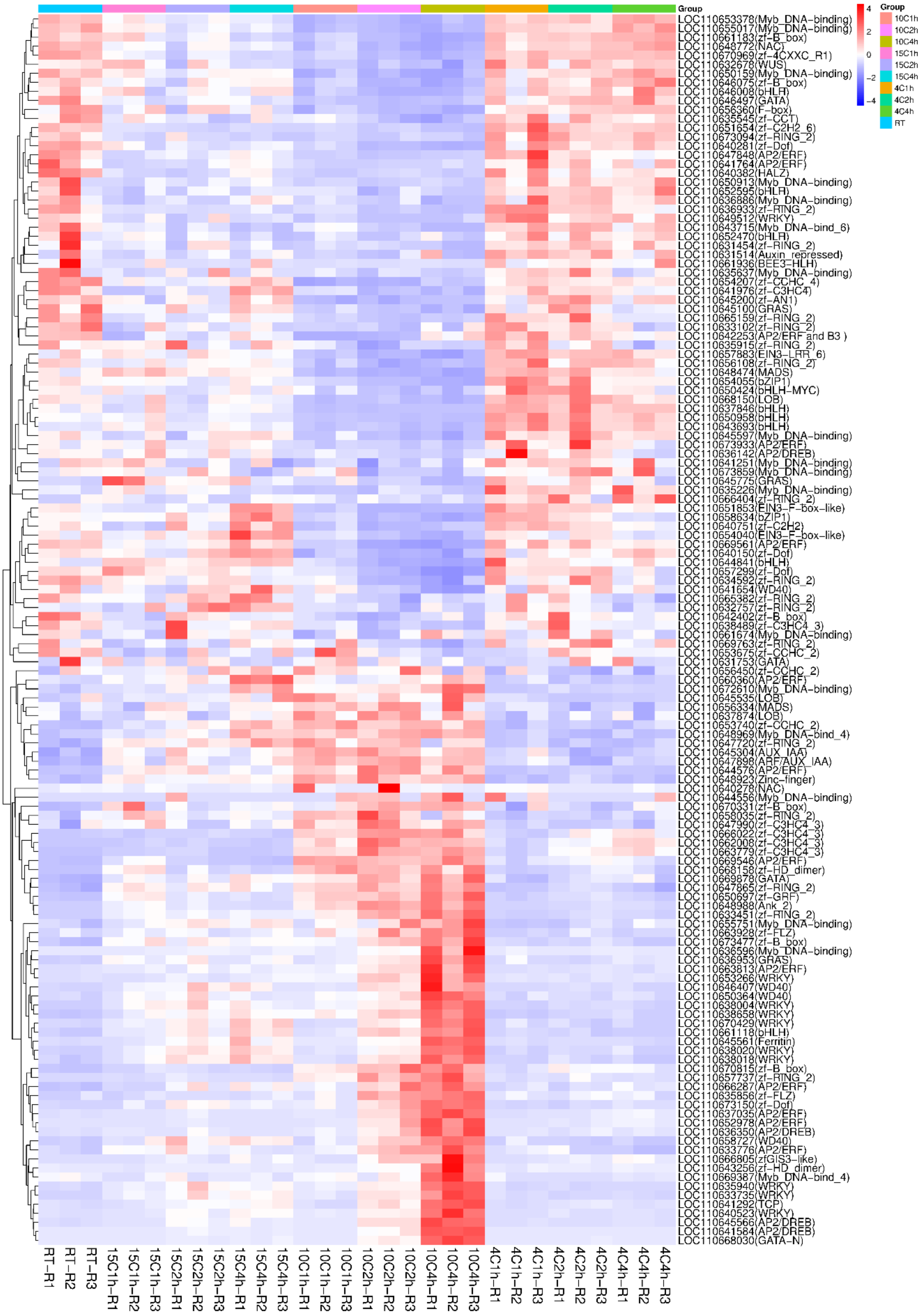

Figure S4. Heatmap of Transcription factors.

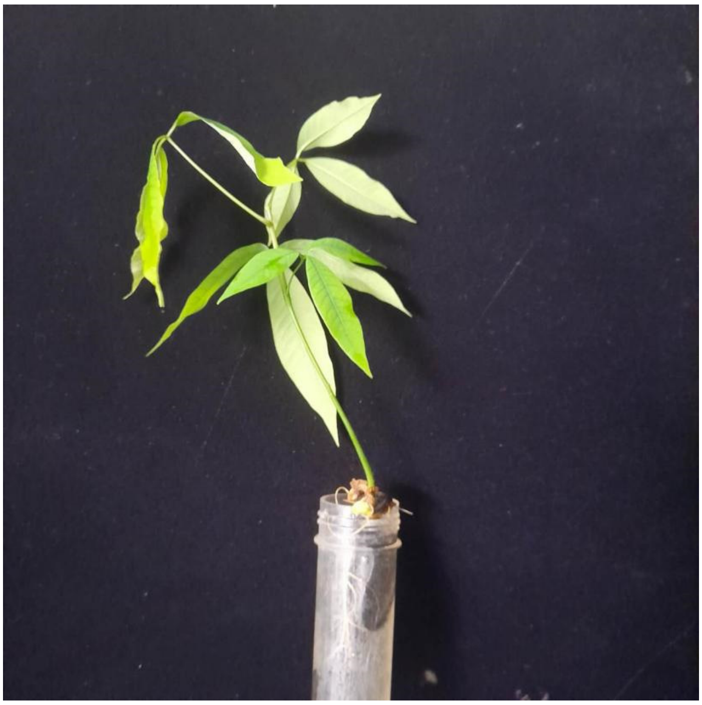

Figure S5. Tissue cultured test tube seedlings of the rubber clone Reyan ‘7-33-97’
