# Supplementary material for: Transcriptomic Analysis of Differential Gene Expression in Hevea brasiliensis Under Short-Term Cold Stress
Source: Plants (Basel). 2025 Sep 18;14(18):2900. doi: 10.3390/plants14182900 (PMC12473355; doi:10.3390/plants14182900)
Supplement: Supplementary file 1 [file plants-14-02900-s001.zip › Volcano plots (Pairwise comparison of different cold treatments).pptx]

## Slide 1
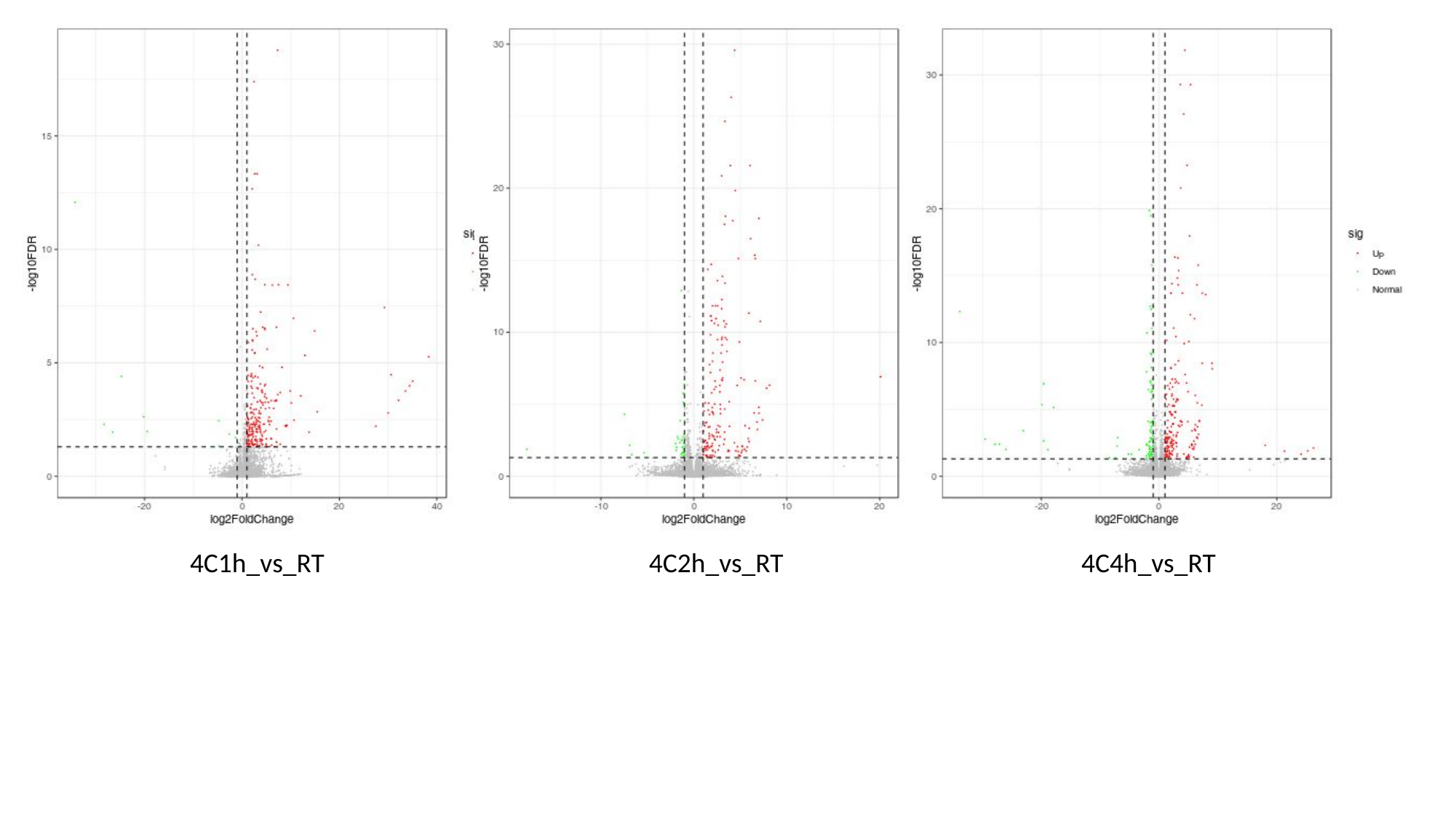

4C1h_vs_RT
4C2h_vs_RT
4C4h_vs_RT

## Slide 2
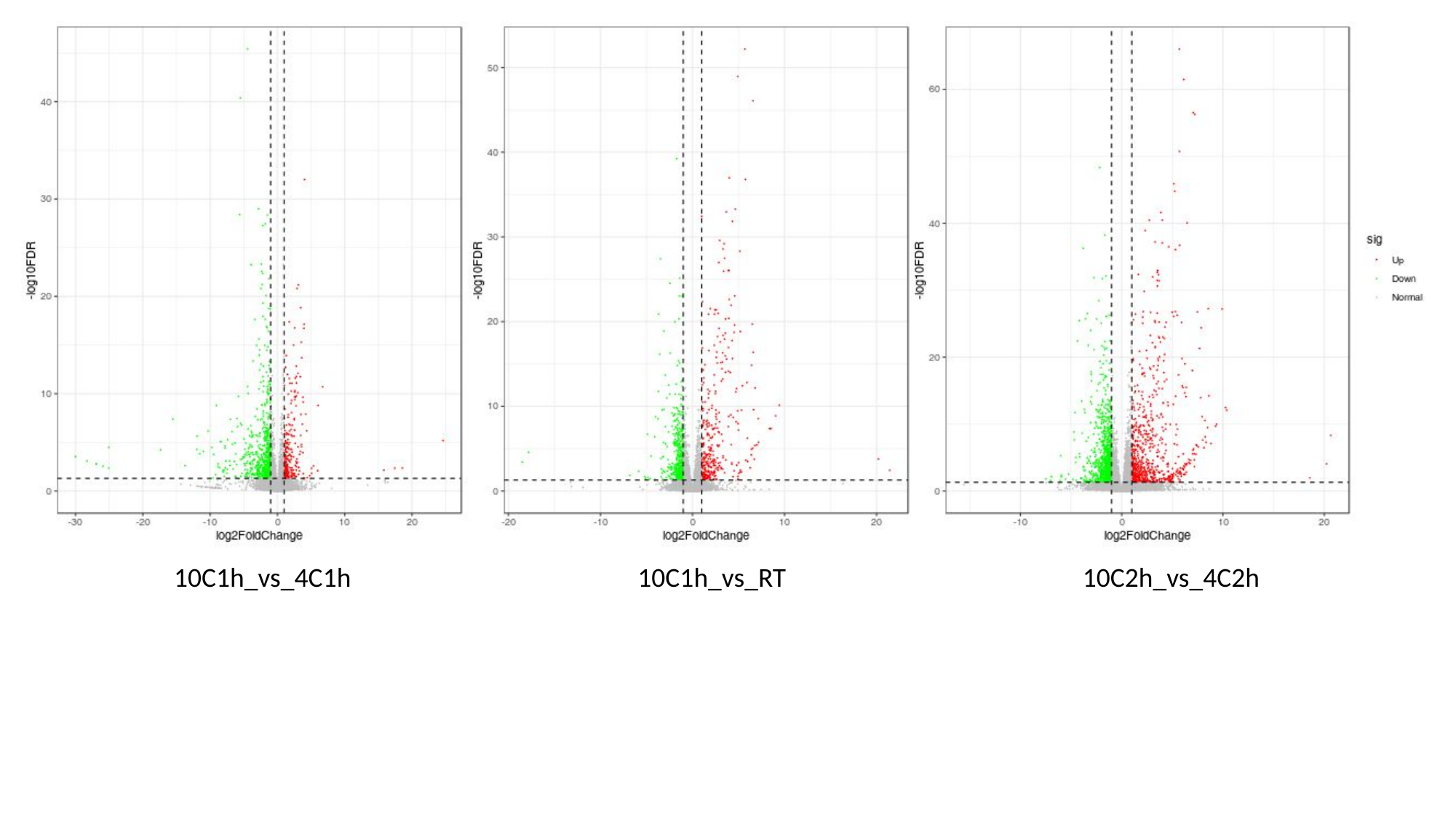

10C1h_vs_4C1h
10C1h_vs_RT
10C2h_vs_4C2h

## Slide 3
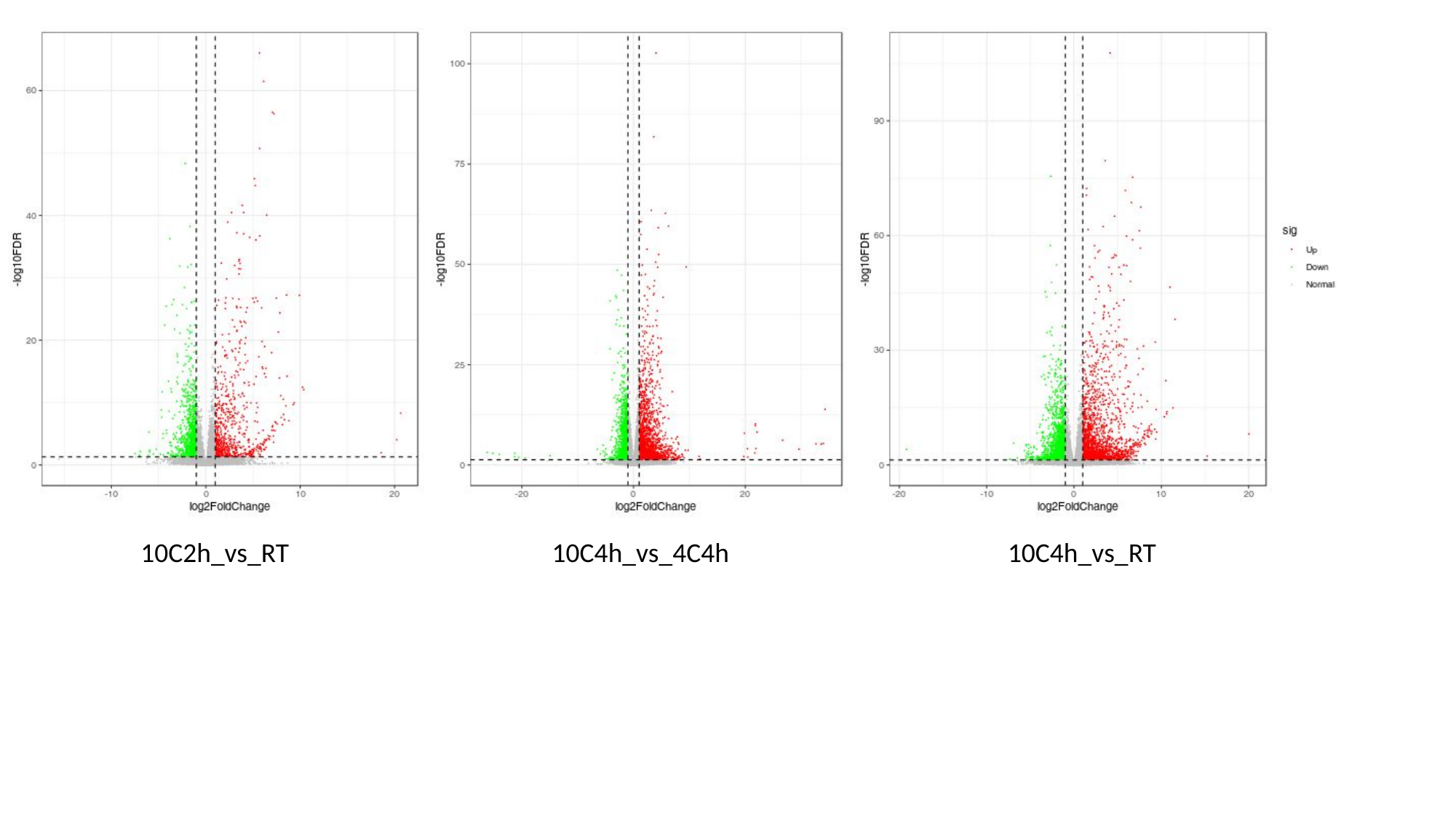

10C2h_vs_RT
10C4h_vs_4C4h
10C4h_vs_RT

## Slide 4
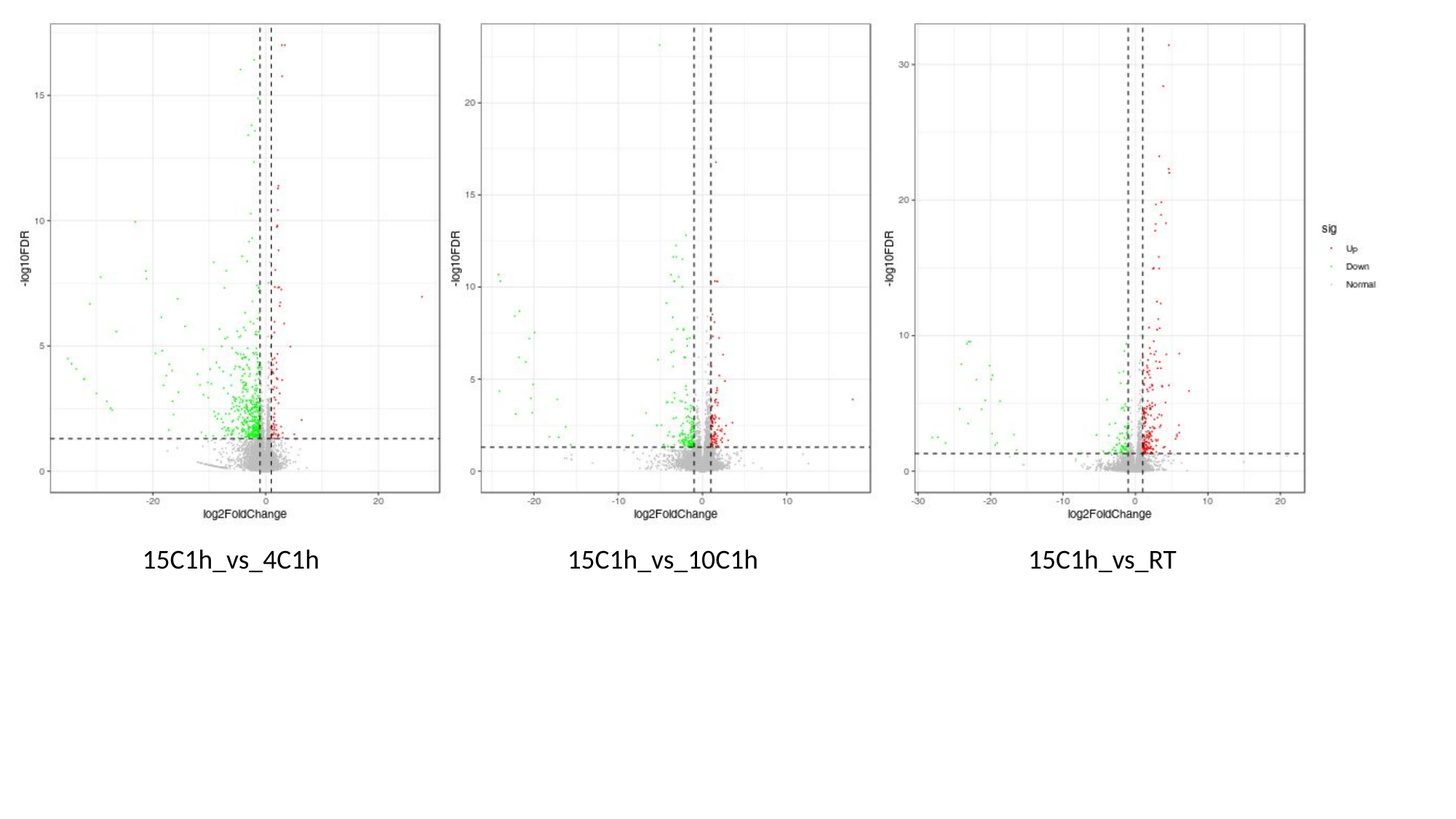

15C1h_vs_4C1h
15C1h_vs_10C1h
15C1h_vs_RT

## Slide 5
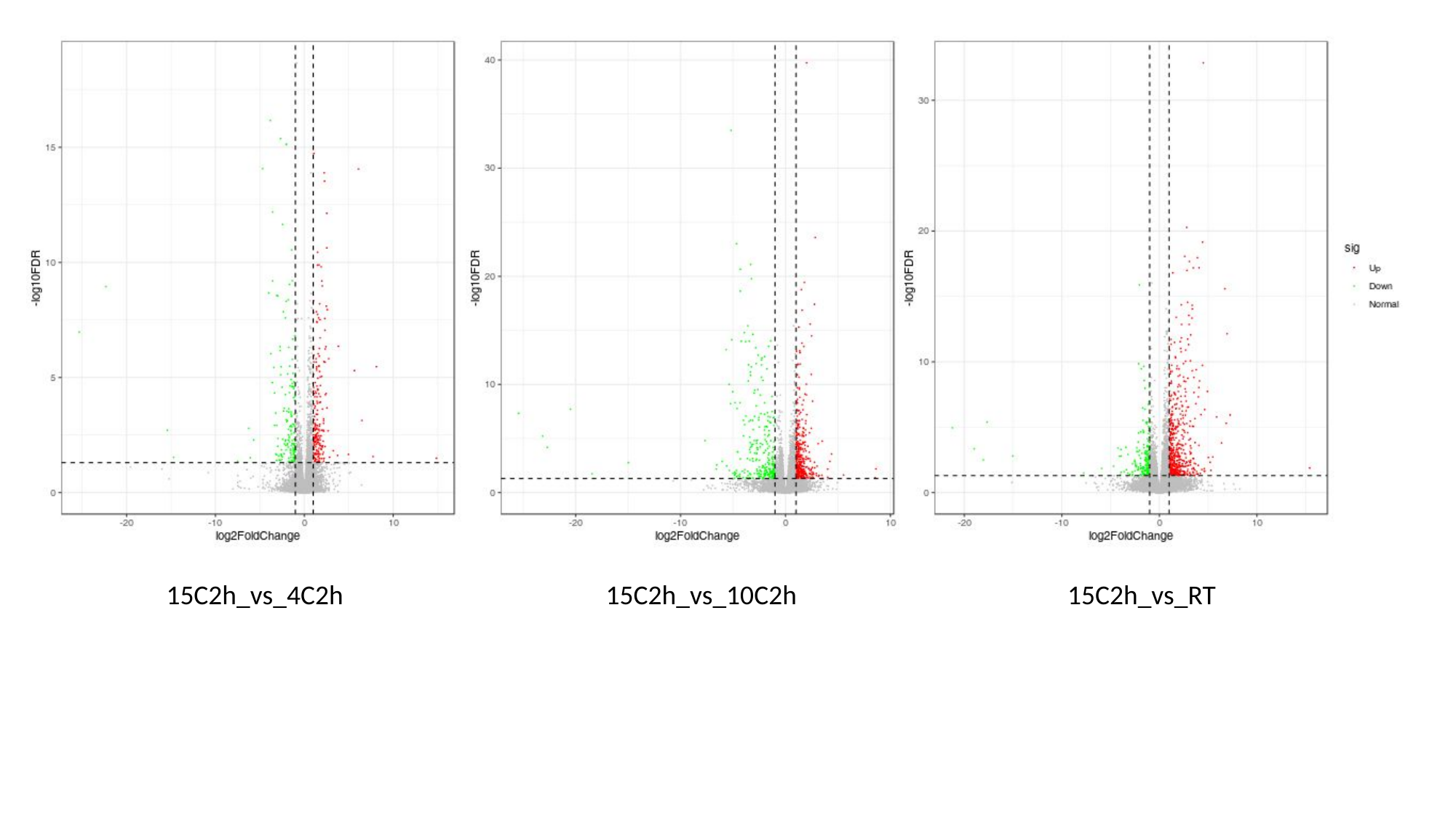

15C2h_vs_4C2h
15C2h_vs_10C2h
15C2h_vs_RT

## Slide 6
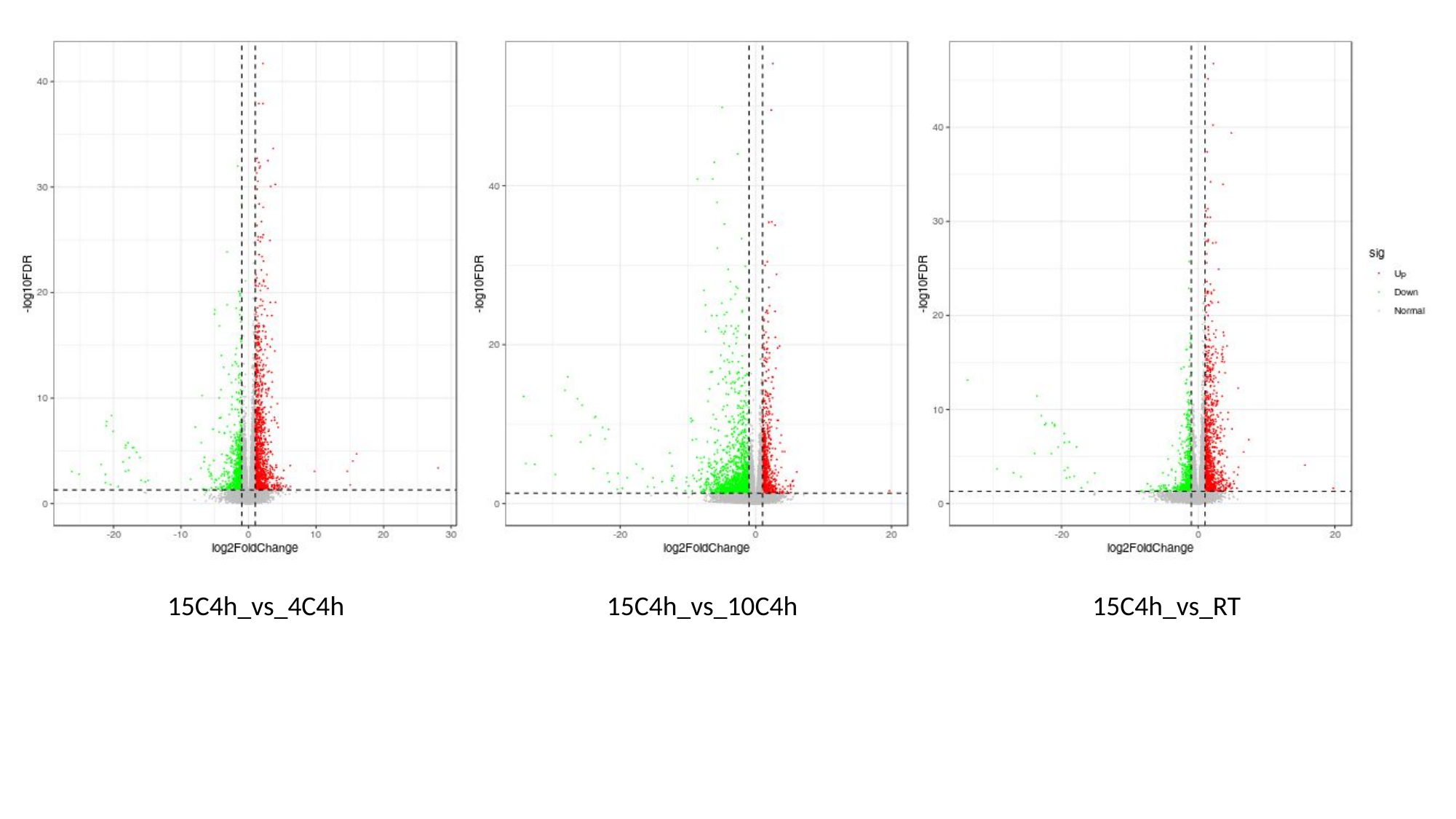

15C4h_vs_4C4h
15C4h_vs_10C4h
15C4h_vs_RT
